# High-resolution QTL mapping for grain appearance traits and co-localization of chalkiness-associated differentially expressed candidate genes in rice

**DOI:** 10.1186/s12284-016-0121-6

**Published:** 2016-09-22

**Authors:** Likai Chen, Weiwei Gao, Siping Chen, Liping Wang, Jiyong Zou, Yongzhu Liu, Hui Wang, Zhiqiang Chen, Tao Guo

**Affiliations:** 1 National Engineering Research Center of Plant Space Breeding, South China Agricultural University, Guangzhou, 510642 China; 2 Guangdong Agricultural Technology Extension, Guangzhou, 510520 China

**Keywords:** Rice, QTL mapping, GBS, Grain shape, Chalkiness, Transcriptome profiling

## Abstract

**Background:**

Grain appearance quality is a main determinant of market value in rice and one of the highly important traits requiring improvement in breeding programs. The genetic basis of grain shape and endosperm chalkiness have been given significant attention because of their importance in affecting grain quality. Meanwhile, the introduction of NGS (Next Generation Sequencing) has a significant part to play in the area of genomics, and offers the possibility for high-resolution genetic map construction, population genetics analysis and systematic expression profile study.

**Results:**

A RIL population derived from an inter-subspecific cross between indica rice PYZX and japonica rice P02428 was generated, based on the significant variations for the grain morphology and cytological structure between these two parents. Using the Genotyping-By-Sequencing (GBS) approach, 2711 recombination bin markers with an average physical length of 137.68 kb were obtained, and a high-density genetic map was constructed. Global genetic mapping of QTLs affecting grain shape and chalkiness traits was performed across four environments and the newly identified stable loci were obtained. Twelve important QTL clusters were detected, four of which were coincident with the genomic regions of cloned genes or fine mapped QTL reported. Eight novel QTL clusters (including six for grain shape, one for chalkiness, and one for both grain shape and chalkiness) were firstly obtained and highlighted the value and reliability of the QTL analysis. The important QTL cluster on chromosome 5 affects multiple traits including circularity (CS), grain width (GW), area size of grain (AS), percentage of grains with chalkiness (PGWC) and degree of endosperm chalkiness (DEC), indicating some potentially pleiotropic effects. The transcriptome analysis demonstrated an available gene expression profile responsible for the development of chalkiness, and several DEGs (differentially expressed genes) were co-located nearby the three chalkiness-related QTL regions on chromosomes 5, 7, and 8. Candidate genes were extrapolated, which were suitable for functional validation and breeding utilization.

**Conclusion:**

QTLs affecting grain shape (grain width, grain length, length-width ratio, circularity, area size of grain, and perimeter length of grain) and chalkiness traits (percentage of grains with chalkiness and degree of endosperm chalkiness) were mapped with the high-density GBS-SNP based markers. The important differentially expressed genes (DEGs) were co-located in the QTL cluster regions on chromosomes 5, 7 and 8 affecting PGWC and DEC parameters. Our research provides a crucial insight into the genetic architecture of rice grain shape and chalkiness, and acquired potential candidate loci for molecular cloning and grain quality improvement.

**Electronic supplementary material:**

The online version of this article (doi:10.1186/s12284-016-0121-6) contains supplementary material, which is available to authorized users.

## Background

The production and consumption of rice is concentrated in Asia where more than 90 % of the world’s rice is grown and consumed (Muthayya et al. 2014; Kong et al. 2015; Jones and Sheats 2016). Appearance (including grain shape and endosperm chalk), cooking properties and texture time were the most important traits affecting grain quality (Fitzgerald et al. 2009). As one of the major aspects of grain quality, grain appearance affects market demand significantly (Tanabata et al. 2012). Even though preferences relating to grain quality properties vary across countries and regions (Calingacion et al. 2014; Concepcion et al. 2015), consumers typically desire rice with uniform shape and translucent endosperm, therefore the quality of appearance directly affects consumer acceptance (Zhao et al. 2015). Grain shape and chalkiness have attracted significant attention in rice genetic research, however, as a practical matter, grain appearance quality is mostly conditioned by quantitative trait locus QTL, representing a major problem for rice improvement programs and production.

Grain shape, widely accepted as a complex quantitative trait, is usually measured as grain length, width, thickness and length-to-width ratio (Bai et al. 2010). Furthermore, digital imaging technology was introduced for computational methods, which could enable us to automatically measure the grain shape parameters of circularity, seed area and perimeter length, etc. (Tanabata et al. 2012). Over the past thirty years, QTL mapping and association analysis have become widely used for analysis of grain appearance traits (Bai et al. 2010; Han and Huang 2013; Huang et al. 2013). By utilizing a variety of mapping populations, such as F_2_, recombinant inbred lines (RILs), backcross and doubled haploid (DH), many QTLs associated with these traits have been identified (Huang et al. 2013). Bai et al. (2010) identified 28 QTLs related to grain shape using a RIL population derived from the cross between japonica and indica rice, and suggested that a mapping population derived from two contrasting parents in grain shape is expected to give rise to a larger number of QTLs. By using map-based cloning strategies, several valuable genes regulating grain shape have been isolated, including *GS3* (Fan et al. 2006), *GW2* (Song et al. 2007), *GS5* (Weng et al. 2008), *qSW5* (Shomura et al. 2008), *OsSPL16* (Wang et al. 2012), *qGL3.1/qGL3* (Qi et al. 2012; Zhang et al. 2012), *GS6* (Sun et al. 2013), *GW7* (Wang et al. 2015a), *SLG7* (Zhou et al. 2015b) and *GL7* (Wang et al. 2015b), which have enhanced our knowledge of the molecular regulatory mechanisms responsible for grain shape and enables breeders to develop high-yield varieties with improved grain-quality (Wang et al. 2015b).

Chalkiness is the other appearance-related trait that affects consumer acceptance of rice (Fitzgerald et al. 2009). Grain chalk is an important indicator of rice quality evaluation and a highly undesirable quality trait in marketing and consumption of the rice grain (Li et al. 2014b). As a polygenic quantitative trait with complex inheritance pattern, chalkiness is highly influenced by the environment. Thus the genetic basis of grain chalkiness is still poorly understood, even though many QTLs for chalkiness or related components have also been identified (http://www.gramene.org). Peng et al. (2014) mapped multiple QTLs associated with six chalkiness traits (chalkiness rate, white core rate, white belly rate, chalkiness area, white core area, and white belly area) using five populations and suggested that most of the QTLs clustered together and could be detected in different backgrounds. Two loci controlling PGWC were mapped by Zhou et al. (2009), and the *qPGWC-7* was narrowed to a 44-kb region. *Chalk5*, regulating grain chalkiness was isolated by Li et al. (2014b), which encodes a vacuolar H^+^-translocating pyrophosphatase (V-PPase) with PPi hydrolysis and H^+^-translocation activity. Elevated expression of *Chalk5* increases chalkiness of the endosperm by disturbing the pH homeostasis in the endomembrane trafficking system in developing seeds, which affects the biogenesis of protein bodies coupled with a great increase in small vesicle-like structures, thus forming air spaces among endosperm storage substances, resulting in chalky grain (Li et al. 2014b). However, the regulation pathway and interaction mechanisms of rice chalkiness associated genes remain unclear.

For traditional QTL mapping, molecular marker genotyping was time consuming and labor-intensive (Chen et al. 2014a). Low-throughput molecular markers such as simple sequence repeats (SSRs) were the most commonly used for linkage maps construction in rice QTL mapping analysis. They are mostly of low density and not able to provide precise and complete information about the numbers and locations of the QTLs controlling the interesting traits (Yu et al. 2011). Single nucleotide polymorphisms (SNPs) are currently the marker of choice due to their large numbers in virtually all populations of individuals (Kumar et al. 2012) and next-generation sequencing (NGS) has enabled the discovery of numerous SNPs for many plant species. Therefore, high-density genetic maps based on SNP markers are achievable and can be developed to improve the efficiency and accuracy of gene or QTL mapping (Li et al. 2014a). In rice, previous studies have demonstrated that the improved quality and resolution of the linkage map based on sequencing-based SNP has greatly facilitated QTL dissection (Huang et al. 2009; Yu et al. 2011; Gao et al. 2013; Zhang et al. 2015).

In this study, we demonstrate the discrepancy in the morphology and cytological structure of two contrasting genotypes and the phenotypic variance of the grain shape and chalkiness traits across a RIL population. Using NGS, a high-density genetic map was constructed based on the new developed bin markers. The QTLs associated with grain shape and chalkiness were identified under four different environments. Moreover, we performed transcriptome expression profiling and identified differentially expressed genes located in the chalkiness-related QTL regions, providing valuable information for candidate gene verification and dissection of gene regulatory networks affecting rice grain appearance quality.

## Results

### The grain appearance and cytological difference between PYZX and P02428

**Fig. 1:**
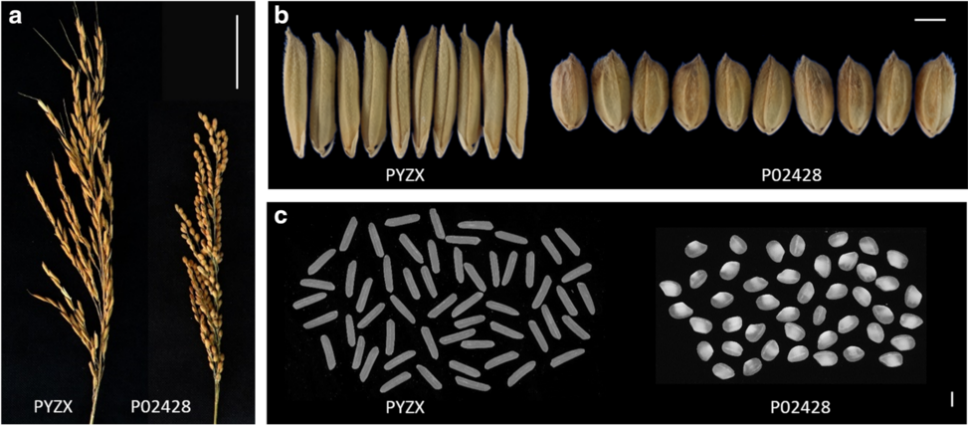
Phenotypic differences between PYZX and P02428 (**a**) panicle (scale bar: 50 mm), (**b**) grain shape (scale bar: 3 mm), and (**c**) milled grains (scale bar: 3 mm)

Considerable distinct variations in panicle structure, grain shape and chalkiness traits between PYZX and P02428 were observed (Fig. 1). PYZX showed much longer and slenderer grain, with limpid kernels, whereas the grain of P02428 was wide and short, along with chalky kernel. The grain appearance traits, including grain length, grain width, the ratio of grain length and width, circularity, area size of grain, and perimeter length of grain for grain shape parameters, and chalk property including percentage of grain with chalkiness and degree of endosperm chalkiness were examined under four environments (Table 1 and Additional file 1: Table S1). The GW and GL of PYZX were at an average of 2.26 mm and 12.17 mm, whereas averages were 3.75 mm and 7.16 mm respectively for P02428. Thus, the LWR of PYZX was almost 3 times the level of P02428, and the CS of the latter was about 2 times greater than the former. Extreme differences in PGWC (0.95 and 91.67 % on avg. for PYZX and P02428 respectively) and DEC (0.09 and 54.16 at avg. for PYZX and P02428 respectively) between parental genotypes were detected continuously across all environments.

**Table 1:** Phenotypic performances and correlation coefficients among the grain shape and chalkiness traits in the RIL population

Phenotypic performances									
Lines		GW (mm)	GL (mm)	LWR	CS	AS (mm^2^)	PL (mm)	PGWC (%)	DEC
PYZX	Mean	3.002	9.826	3.714	0.548	20.848	23.295	44.446	25.334
	S.D	0.043	0.171	0.116	0.003	0.842	0.278	0.460	0.043
P02428	Mean	3.754	7.163	1.837	0.729	19.425	18.644	91.667	54.158
	S.D	0.087	0.225	0.161	0.008	0.642	0.747	4.659	4.192
RILs	Mean	2.743	9.404	3.461	0.516	19.650	21.950	28.260	19.430
	S.D	0.028	0.161	0.026	0.002	0.588	0.359	5.936	7.989
Correlation coefficients									
GW			−0.378^b^	−0.766^b^	0.822^b^	0.557^b^	−0.125^b^	0.556^b^	0.357^b^
GL				0.759^b^	−0.760^b^	0.608^b^	0.919^b^	−0.130^a^	−0.038
LWR					−0.886^b^	−0.047	0.669^b^	−0.462^b^	−0.248^a^
CS						0.043	−0.672^b^	0.455^b^	0.286^b^
AS							0.702^b^	0.374^b^	0.298^b^
PL								−0.049	−0.014
PGWC									0.626^b^

^a^, ^b^ significant at the level of 0.05 and 0.01, respectively

**Fig. 2:**
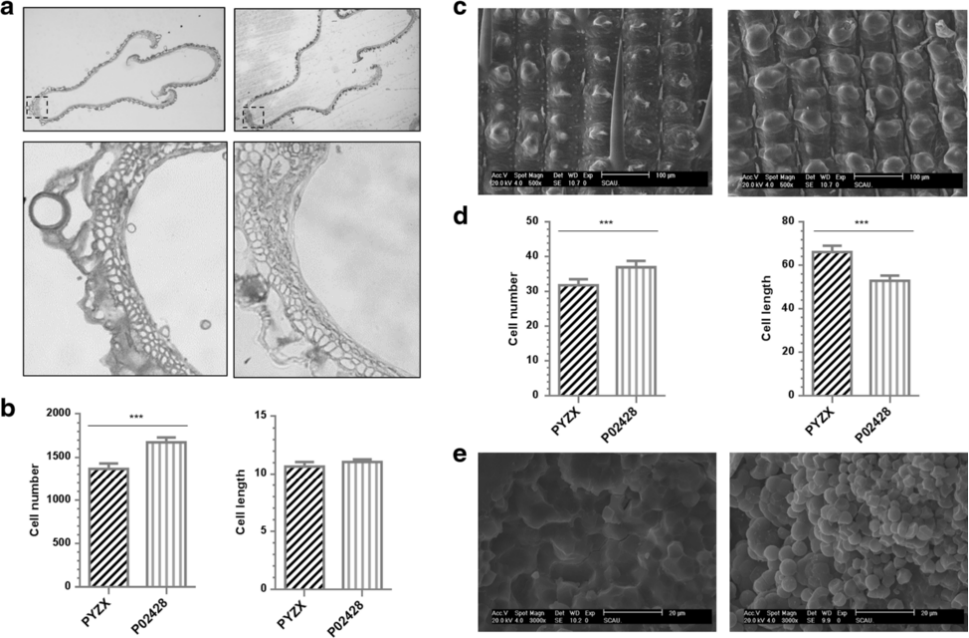
Cellular analyses of spikelet hull and endosperm of PYZX and P02428 grains. **a** Cross-section of spikelet hull. Upper: cross-section of spikelet hull (100×). Dotted line indicates position of cross-section. Lower: magnified view of spikelet hull cross-section. **b** Comparison of total cell number and mean cell length in the cross-section of outer glume cell layers of spikelet hull. **c** Scanning electron microscopy photos of outer glume surfaces (500×). **d** Comparison of total cell number and mean cell length in the outer glume surface of spikelet hull. **e** Scanning electron microscopy images of transverse sections from the endosperm bellies of mature seeds (3000×). ***P < 0.001; Student’s *t* test was used to generate the *P* values in (**b**) and (**d**)

Microscopic observation with a cross-section of spikelets indicated that P02428 contained substantially higher cell numbers when compared to that of PYZX, with only an insignificant increase in cell length (Fig. 2a, b). A scanning electron microscope investigation of outer glume surfaces demonstrated a significant increase in cell numbers and decrease in cell length for P02428 compared to PYZX (Fig. 2c, d). This histological analysis established the major origins of the observed grain shape and size variation between the parental lines. Transverse sections of the endosperm bellies of mature seeds were also examined using scanning electron microscopy, and revealed that the endosperm of chalky grains of P02428 contained loosely packed starch granules with large air spaces, while those of PYZX were filled with densely packed granules (Fig. 2e).

### Phenotypic variation of grain shape and chalkiness parameters in RIL population

**Fig. 3:**
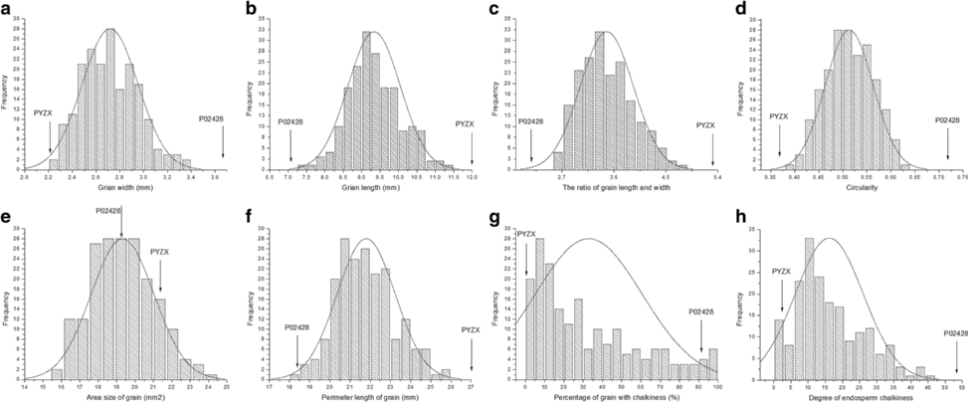
Frequency distribution of grain shape and chalkiness parameters of the RILs population derived from cross between PYZX and P02428. Average trait values of four environments were used. Mean phenotypic values of both parental lines are indicated by arrows. **a** GW, (**b**) GL, (**c**) LWR, (**d**) CS, (**e**) AS, (**f**) PL, (**g**) PGWC and (**h**) DEC

Generally, the RIL population exhibited an extremely wide variation in rice grain shape and chalkiness traits and continuous distributions were observed for all eight investigated traits (Fig. 3), consistent with quantitative traits controlled by multi-genes. All of the grain shape related parameters were evenly distributed as single peak patterns, whereas the phenotypic values of PGWC and DEC were exhibited in the specific asymmetric distributed pattern. Furthermore, PGWC and DEC for the RIL population also showed higher standard deviations (Table 1), indicating that they are significantly affected by environments. For GW, GL, LWR and CS, the average value of traits measured in the RIL population was between the two parental lines, and none of the individual lines exhibit values that surpass either P02428 or PYZX. A large amount of variation and the greatest transgressive segregation was observed for the trait of AS.

The correlation among the grain shape and chalkiness parameters in the RIL population was analyzed (Table 1). The results showed that significant correlations were detected between grain shape and chalkiness trait. PGWC and DEC were positively correlated with GW, CS, and AS, while negatively correlated with LWR. The correlation coefficient between PGWC and DEC was high (*r* = 0.626). For grain shape traits, CS, AS, and PL were significantly correlated with GW and GL concurrently. We also found a considerably high positive correlation between LWR and CS (*r* = 0.886).

### Genotyping by sequencing and bin markers establishment

In this study, a total of 83.88 Gb high-quality sequence data from 559,213,384 pair-end reads was obtained and about 97.48 % of those reads were mapped to the Nipponbare reference genome. For 192 RILs individuals, total mapped regions covered by the captured fragments were about ~7.0 % of the genome sequence and with a coverage depth of ~11.76× on average for the captured regions. Initial analysis identified 1,534,036 SNPs between the two parents (see Additional file 1: Table S2 for annotation statistics of location) of which 1,334,454 were consistent with the “aa × bb” type (presence of polymorphism between the genotypes of parents and both of them were homozygous) were selected for further analysis. Genotypes of the RIL individuals at these SNP sites were determined, and 123,982 loci with more than 4 base depth remained. After filtering for abnormality, a total of 85,742 high-quality SNPs were validated for recombinant event determination.

**Fig. 4:**
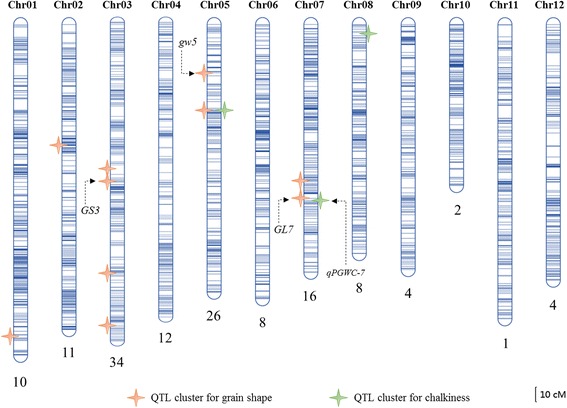
Genetic linkage map constructed with bin markers and location of QTLs associated with the grain shape and chalkiness traits. Blue lines represent the positions of bin markers on each linkage group. QTL clusters are indicated on the chromosome corresponding to their genetic position. The total numbers of QTL detected per chromosome are shown below the chromosome. Detailed information on these QTLs is in Additional file 1: Table S3

Consecutive SNPs were examined (with a sliding window size of 15 SNPs) and the same genotypes were lumped into recombination bins. Bins with an interval of less than 300 kb and the number of sequenced SNPs fewer than five were masked as missing data to avoid false double recombinations (Xie et al. 2010). Adjacent bins of the same genotype across the entire RIL population were merged and transition between two different genotype bins was determined as a breakpoint. After this processing, a total of 2711 recombination bin markers along the 12 chromosomes were adopted to construct a bin map for the RIL population (Fig. 4). The average physical length between the recombination bin markers was 137.68 kb, and the average annotated gene loci among the markers ranged from 15.34 for Chr04 to 22.09 for Chr01 (Table 2). Using the R/qtl package (est.map function with Lander-Green algorithm), we constructed a genetic linkage map with an average distance of 0.86 cM between adjacent bin markers and a maximum spacing between markers ranging from 9.93 cM for Chr03 to 5.84 cM for Chr10. The average genetic size of the 12 chromosomes is about 195.31 cM, and the average physical distance between markers was 137.68 Kb (Table 2).

**Table 2:** Distribution of genetic markers across the 12 chromosomes in rice

Chromosome	Number of bin markers	Length (cM)	Average genetic distance between markers (cM)	Maximum spacing between markers (cM)	Average physical distance between markers (Kb)	Average number of annotated gene loci among markers^a^
1	295	232.15	0.79	8.25	146.68	22.09
2	258	221.65	0.86	7.20	139.29	20.90
3	311	226.44	0.73	9.93	117.09	18.66
4	278	223.22	0.80	8.53	127.71	15.34
5	182	194.90	1.07	6.85	164.61	22.03
6	229	199.68	0.87	9.75	136.46	17.31
7	232	180.72	0.78	6.50	128.01	16.24
8	179	167.37	0.94	6.51	158.90	19.14
9	178	178.69	1.00	7.19	129.28	15.55
10	162	118.78	0.73	5.84	143.25	17.47
11	208	213.82	1.03	9.37	139.52	15.42
12	199	186.26	0.94	9.37	138.35	14.99
Overall	2711	2343.68	0.86	9.93	137.68	18.12

^a^ annotation on Os-Nipponbare-Reference-IRGSP-1.0 (http://rapdb.dna.affrc.go.jp/)

### Comprehensive QTL mapping for the grain shape and chalkiness traits

With the inclusive composite interval mapping method, a total of 136 loci affecting grain shape and chalkiness traits were detected according to the LOD threshold across four environments (47 for G-DS, 28 for Z-DS, 38 for G-WS and 23 for Z-WS respectively) (Additional file 1: Table S3 and Fig. 4). The majority of the QTLs associated with grain width and circularity traits had a negative additive effect, indicating that alleles from the parent P02428 contributed to increasing phenotype, whereas the phenotypes of GL, LWR and PL were mainly contributed by PYZX. For AS traits, 62.5 and 37.5 % of the QTLs had a negative and positive effect respectively, indicating both parents contributed favorable alleles. All of the QTLs related to chalkiness were endowed with the additive effect contributed by P02428 (Additional file 1: Table S3).

Seventeen QTLs were detected for GW and each QTL explained 3.354 ~ 12.377 % of the phenotypic variation. Among all QTLs identified for GW, five QTLs showed high PEV value of more than 10 %, including three major QTLs on Chr05 and two on Chr07. Sixteen QTLs were detected for GL, explaining 3.904 ~ 20.799 % of phenotypic variation for each QTL, and six showed major QTL with higher LOD and effect value, located on Chr03 and Chr07, remarkably similar to that of the PL and LWR parameter. QTLs associated with AS were also identified in these regions as well and a QTL with LOD of 7.044 and PEV of 10.657 % was detected on Chr10. Twenty-four QTLs for CS parameter were detected across ten chromosomes using single-environment analysis and each QTL explained 3.403 ~ 26.139 % of phenotypic variation, and six major QTLs with higher PEV value were located on chromosomes 3, 4, 5 and 7. There were ten QTLs for DEC and each QTL explained 5.391 ~ 12.779 % of phenotypic variation, and sixteen QTLs associated with PWGC were detected explaining 4.457 ~ 12.975 % of phenotypic variation. More than 81 % of the QTLs for PGWC and DEC provided a lower PEV value, indicating that the chalkiness-related parameters were regulated with polygene and minor effects. However, four major genomic regions harboring QTLs with higher LOD and PEV values for PGWC or DEC were identified, and more importantly, six common QTL regions were detected for both PGC and DEC and distributed on chromosomes 4, 5, 7, and 8 (Table 3).

**Table 3:** QTLs associated with chalkiness traits detected in the different environments

Trait	Chromosome	Peak Position (cM)	Interval (cM)		Left Marker	Right Marker	LOD	PVE (%)^a^	Add^b^	Environment
PGWC	2	168	167.52	169.65	mk473	mk475	3.165	5.594	−7.291	Z-DS
	4	113	111.78	113.37	mk998	mk1000	7.035	12.975	−10.997	Z-DS
	4	135	134.51	136.37	mk1005	mk1007	5.635	9.738	−9.579	G-DS
	5	13	9.76	14.4	mk1153	mk1155	2.638	4.971	−8.125	G-WS
	5	35	34.39	35.18	mk1174	mk1175	5.250	8.985	−8.999	G-DS
	5	60	56.09	61.75	mk1189	mk1191	6.347	11.890	−10.337	Z-DS
	5	60	56.09	61.75	mk1189	mk1191	4.007	8.143	−9.706	G-WS
	5	84	83.81	85.4	mk1228	mk1230	2.773	5.182	−8.332	G-WS
	6	55	54.62	56.51	mk1378	mk1379	3.002	5.819	−8.807	G-WS
	6	78	77.75	78.01	mk1408	mk1409	2.568	4.457	−6.255	Z-DS
	7	132	130.52	132.94	mk1743	mk1745	3.662	7.123	−7.934	Z-DS
	7	134	133.2	134.53	mk1746	mk1747	2.946	4.763	−6.593	G-DS
	8	1	0.53	1.85	mk1786	mk1789	4.804	9.866	−10.693	Z-WS
	8	4	1.85	3.46	mk1789	mk1791	4.076	6.701	−7.893	G-DS
	8	17	16.85	17.11	mk1803	mk1804	4.580	8.645	−10.740	G-WS
	9	108	107.39	108.72	mk2078	mk2079	2.642	5.430	−7.888	Z-WS
DEC	4	110	109.95	110.21	mk991	mk992	3.016	6.342	−3.745	Z-WS
	4	136	134.51	136.37	mk1005	mk1007	3.779	7.760	−2.188	G-DS
	5	18	17.86	21.8	mk1160	mk1161	3.589	5.831	−4.473	G-WS
	5	35	34.39	35.18	mk1174	mk1175	3.354	6.847	−2.014	G-DS
	5	60	56.09	61.75	mk1189	mk1191	3.660	8.462	−1.813	Z-DS
	5	61	56.09	61.75	mk1189	mk1191	3.516	7.464	−4.003	G-WS
	6	23	22.67	23.19	mk1339	mk1340	2.516	5.391	−3.390	Z-WS
	6	64	63.57	65.46	mk1387	mk1388	7.378	12.779	−6.611	G-WS
	7	134	133.2	134.53	mk1746	mk1747	3.610	6.532	−0.015	Z-WS
	8	6	5.87	8.33	mk1792	mk1793	6.002	10.074	−5.931	G-WS
	8	130	129.72	130.24	mk1924	mk1925	3.411	7.027	−2.046	G-DS

^a^ phenotypic variation explained; ^b^ additive effects

### Stable QTLs and major QTL clusters

As many of the QTLs detected here overlapped, they were classified into same loci according to the genetic position. Three or more QTLs detected for the same trait within the consistent confidence interval using single environment analysis were defined as stable in this study. Notably, most of these QTLs had good reproducibility across multiple environments. For instance, 54.17 % of the grain circularity QTLs were reproducible under the various environments. The main effect QTL located on Chr03 between mk690 ~ mk698 was detected up to four times, and the QTL located in the genetic interval between mk1743 ~ mk1747 were stably reproducible in three separate environments (Additional file 1: Table S3). QTLs associated with different traits located within the same confidence marker intervals were grouped together as major QTL clusters. As a result, we concluded that there were twelve QTL clusters distributed over six chromosomes (Table 4). Some of the traits with high inter-trait correlations appeared to cluster together, which was in accordance with our correlation analysis between the traits (Table 1), revealing the main genetic determinants of grain shape and chalkiness characteristics in rice. Among these, the QTL cluster of *qGS7.2* (mk1743-mk1745) was identified as the major grain shape QTL explaining highest phenotypic variance in our study (Table 4). The previously reported QTL of *GL7*, which overlaps with this QTL cluster region, encodes a protein homologous to *Arabidopsis thaliana* LONGIFOLIA proteins and was reported to regulate grain appearance quality mainly by affecting the grain length to width ratio and the formation of starch granules in endosperm (Wang et al. 2015b). In addition, three other QTL clusters for grain shape or chalkiness traits were mapped to relatively narrow genomic regions that coincided with QTLs in previously published reports including *GS3* (Fan et al. 2006; Mao et al. 2010), *gw5/qSW5* (Shomura et al. 2008; Weng et al. 2008), and *qPGWC-7* (Zhou et al. 2009) (Table 4), thus supporting the accuracy of our linkage map and mapping analysis.

**Table 4:** Major QTL clusters associated with grain shape and chalkiness traits detected in this study

QTL cluster	Chromosome	Marker interval	Physical interval (100 kb)	Involved traits	LOD	PVE (%)^a^	Overlapped QTL reported
*qGS1*	1	mk289-mk295	425.5–432.5	GW, LWR and CS	3.122–4.948	3.781–7.943	
*qGS2*	2	mk401-mk405	162.5–167.5	GL, LWR and PL	2.765–4.789	3.540–8.689	
*qGS3.1*	3	mk686-mk692	156.5–162.5	AS, PL, CS, GL and LWR	3.161–12.450	7.228–18.648	
*qGS3.2*	3	mk693-mk698	163.5–169	CS, GL, LWR and PL	3.151–14.296	5.068–21.024	*GS3* (Fan et al. 2006)
*qGS3.3*	3	mk794-mk795	286.5–287.5	CS, GL, LWR and PL	2.648–5.080	4.176–6.470	
*qGS3.4*	3	mk819-mk822	311.5–314.5	GL and PL	2.921–5.690	5.600–8.828	
*qGS5.1*	5	mk1174-mk1175	51.5–55.5	CS, GW and LWR	3.047–8.912	3.729–11.617	*gw5* (Weng et al. 2008)
							*qSW5* (Shomura et al. 2008)
*qGS5.2, qPGWC5* (*qDEC5*)	5	mk1189-mk1192	76.5–79.5	CS, GW and AS; PGWC and DEC	2.686–6.347	6.388–12.377	
*qGS7.1*	7	mk1734-mk1739	232.5–239	CS, LWR, GL and PL	4.297–11.568	8.141–17.573	
*qGS7.2*	7	mk1743-mk1745	244–248	CS, GW, LWR and GL	18.346–18.346	12.326–26.139	*GL7* (Wang et al. 2015a, b)
							*GW7* (Wang et al. 2015a)
							*SLG7* (Zhou et al. 2015b)
*qPGWC7* (*qDEC7*)	7	mk1743-mk1747	244–251.5	PGWC and DEC	2.946–3.662	4.763–7.123	*qPGWC-7* (Zhou et al. 2009)
*qPGWC8* (*qDEC8*)	8	mk1786-mk1793	1.5–7.5	PGWC and DEC	4.076–6.002	6.701–10.074	

^a^ phenotypic variation explained

Significantly, the other eight novel QTL clusters detected in this study (Table 4) contributed the stable effects on the phenotype across different environments which underscores the value and reliability of the QTL analysis. The QTL cluster of *qGS5.2, qPGWC5* (*qDEC5*) (mk1189-mk1192) on Chr05 (Table 4 and Fig. 4) simultaneously affected the traits of CS, GW, AS, PGWC and DEC and explained a phenotypic variation of 6.388 ~ 12.378 % for each trait, suggesting some pleiotropic effect for this QTL cluster. The *qGS5.2* allele for increasing grain CS, GW and AS was contributed by the parent P02428, which also contributed the *qPGWC5* (*qDEC5*) allele for increased PGWC and DEC. These results strongly suggest that *qGS5.2* and *qPGWC5* (*qDEC5*) represent the same locus controlling both grain shape and chalkiness. Around this QTL region, Gao et al. (2015) also roughly mapped five major QTLs (the interval was large) affecting GW, LWR, GT (grain thickness), PGWC, and TGW (1000-grain weight), each explaining up to 44.30, 55.29, 62.30, 30.94, and 28.78 % of the variation. Consequently, this QTL cluster could be a novel genetic region controlling multiple grain quality traits.

The QTL clusters of *qGS3.1* and *qGS7.1* associated with grain shape on Chr03 and Chr07 harbor more than four QTL loci, showing high stability and PVE value in multiple environments. Whereas the *qGS3.3* was extrapolated to be a minor-effect QTL, and allele of P0428 contributed positively to grain shape (GL, CS, LWR and PL). *qPGWC8* (*qDEC8*), which has not been reported previously, was identified for both PGWC and DEC, explaining the phenotypic variation of 6.70 ~ 10.07 %. In addition, there were three other QTL clusters with relatively smaller effects: *qGS1* (QTLs for GW, LWR and CS), *qGS2* (QTLs for GL, LWR and PL), and *qGS3.4* (QTLs for GL and PL). Due to high correlations between the examined traits, it is highly likely that these loci have pleiotropic effects on multiple characters, rather than closely linked loci affecting individual characters. These QTL intervals were assumed to harbor novel gene loci affecting grain shape or chalkiness traits, and therefore are worth further investigating.

### Gene expression profile and identification of candidate genes associated with chalkiness

**Fig. 5:**
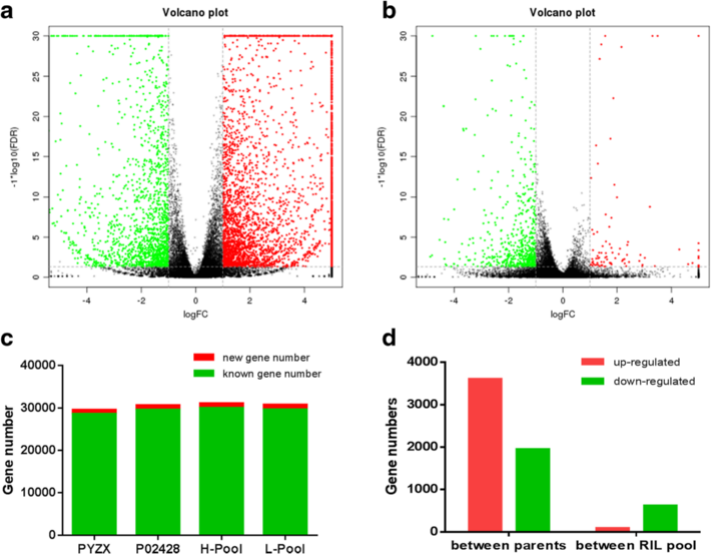
Transcriptome profiling of parents and bulked RILs. **a** Differentially expressed genes between parents, (**b**) Differentially expressed genes between bulked RILs, (**c**) Gene number mapped across four sequencing library, (**d**) Up and down regulated genes detected between different samples

To investigate the gene regulation patterns during grain development and perform a large-scale inspection of different expressed genes (DEGs) correlated with the chalkiness traits, RNA-Seq analysis was performed on the parental lines and two bulked pools made of RILs exhibiting extreme PGWC and DEC phenotypes. The total number of genes detected in each of the bulks is shown in Fig. 5c. After a significance test, 3603 genes with increased expression and 1949 genes with decreased expression were identified for P02428 compared to those of PYZX (Additional file 2: Table S4, Fig. 5a, d). Such a large number of differentially expressed genes could not be responsible for the variation in chalkiness traits between PYZX and P02428. Normalization to remove the background noise of DEGs not related to chalkiness resulted in the identification of 88 genes with increased expression and 623 genes with decreased expression in the L-Pool (pool with extremely low levels of PGWC and DEC) compared to the H-Pool (pool with high levels of PGWC and DEC) (Additional file 3: Table S5, Fig. 5b, d). Functional annotations of the DEGs were analyzed and the investigation of GO enrichment (Gene Ontology Enrichment) was performed (Additional file 2: Table S4, Additional file 3: Table S5, and Additional file 4: Figure S1).

We analyzed the region bound by the flanking markers and co-located these DEGs near the QTL regions for PGWC and DEC including cluster 8, 11 and 12. A total of thirty-three DEGs were co-located in these three QTL regions (Table 5). Most were expressed more highly in H-Pool than in L-Pool and were higher in P02428 than in PYZX. Three genes with significantly different expression were located at the *qPGWC5* (*qDEC5*) locus: the bidirectional sugar transporter SWEET3a (*Os05g0214300*), the UDP-glucuronosyl/UDP-glucosyltransferase family protein (*Os05g0215300*), and the class III peroxidase 73 (*Os05g0231900*) (Table 5). qRT-PCR analysis indicated that the expression level of SWEET3a, a gene involved in sugar transport, was about fifteen times higher in the H-Pool than in the L-Pool. This gene was also strongly upregulated in P02428 compared to PYZX in grain tissue. *Os05g0215300* was expressed 4.55 fold higher in the H-Pool than comparing to L-Pool. The remaining thirty DEGS were located at *qPGWC7* (*qDEC7*) (17) and *qPGWC8* (*qDEC8*) (13) (Table 5). Of particular interest, the UDP-arabinopyranose mutase 3 (*Os07g0604800*) gene which was up-regulated in P02428 is located in the *qPGWC7* (*qDEC7*) region and beta-glucosidase, GBA2 type domain containing protein (*Os08g0111200*) and fructose-bisphosphate aldolase (*Os08g0120600*) mapped to the *qPGWC8* (*qDEC8*) region. Differential expression of these genes was also validated by qRT-PCR and the results of the two experiments were basically consistent. These genes are the most suitable candidates for molecular cloning and development of new functional gene-target markers to facilitate marker assisted breeding.

**Table 5:** Annotated function of differentially expressed genes identified within or near the QTLs affecting chalkiness trait

No.	Gene ID	H-Pool vs. L-Pool					PYZX vs. P02428				Symbol	Description
		Fold change	log_2_(FC)	FDR	Sig.	Fold change (qRT-PCR)	Fold change	log_2_(FC)	FDR	Sig.		
*qPGWC5* (*qDEC5*)												
1	*Os05g0214300*	(−)11.74	−3.554	0.003	yes	(−)15.33	(+)430.00	8.748	0.029	yes	*OsSWEET3a*	Similar to Bidirectional sugar transporter SWEET3a.
2	*Os05g0215300*	(−)2.07	−1.046	0.000	yes	(−)4.55	(+)22.53	4.494	0.000	yes		UDP-glucuronosyl/UDP-glucosyltransferase family protein.
3	*Os05g0231900*	(+)2.85	1.511	0.013	yes		(−)1263.33	−10.303	0.009	yes	*prx73*	Hypothetical conserved gene.
*qPGWC7* (*qDEC7*)												
1	*Os07g0597000*	(−)2.24	−1.163	0.000	yes		(+)1.84	0.878	0.000	no		Similar to Eukaryotic translation initiation factor 5A (eIF-5A).
2	*Os07g0597050*	(−)2.78	−1.473	0.000	yes		(+)6.30	2.654	0.000	yes		Hypothetical gene.
3	*Os07g0597100*	(−)2.62	−1.392	0.000	yes		(+)5.74	2.522	0.000	yes		Similar to Saccharopine dehydrogenase.
4	*Os07g0597400*	(−)7.63	−2.931	0.000	yes		(+)168.27	7.395	0.000	yes		Conserved hypothetical protein.
5	*Os07g0599300*	(−)4.27	−2.094	0.033	yes		(+)15.84	3.986	0.002	yes		Hypothetical protein.
6	*Os07g0599500*	(−)3.73	−1.900	0.004	yes		(+)2483.33	11.278	0.000	yes		Hypothetical protein.
7	*Os07g0599600*	(−)4.39	−2.135	0.000	yes		(+)6.29	2.652	0.000	yes		Hypothetical protein.
8	*Os07g0599700*	(−)3.69	−1.885	0.000	yes		(+)3.88	1.955	0.000	yes		Similar to Surface protein PspC.
9	*Os07g0599900*	(−)6.15	−2.620	0.000	yes		(+)14.53	3.861	0.000	yes		Conserved hypothetical protein.
10	*Os07g0600300*	(−)2.67	−1.415	0.005	yes		(+)2.22	1.153	0.052	no		Protein of unknown function DUF794, plant family protein.
11	*Os07g0601100*	(−)6.04	−2.594	0.000	yes		(+)4.60	2.201	0.000	yes	*DHFR*	Similar to NADPH HC toxin reductase (Fragment).
12	*Os07g0602000*	(−)2.21	−1.142	0.001	yes		(+)26.60	4.733	0.000	yes	*DHFR*	Similar to NADPH HC toxin reductase (Fragment).
13	*Os07g0602900*	(−)4.42	−2.144	0.001	yes		(+)1.75	0.805	0.029	no		Protein of unknown function DUF1675 domain containing protein;Similar to UPF0737 protein 1.
14	*Os07g0604800*	(−)3.52	−1.815	0.000	yes	(−)2.63	(+)2.09	1.062	0.000	yes	*OsUAM3*	Similar to Alpha-1,4-glucan-protein synthase [UDP-forming] 1 (EC 2.4.1.112) (UDP- glucose:protein transglucosylase 1) (UPTG 1).
15	*Os07g0607500*	(−)2.09	−1.067	0.049	yes		(+)2.59	1.371	0.035	yes		Protein of unknown function DUF1195 family protein.
16	*Os07g0616750*	(+)2.13	1.090	0.034	yes		(−)2.34	−1.229	0.012	yes		Hypothetical gene.
17	*Os07g0617100*	(−)2.17	−1.120	0.000	yes		(+)1.01	0.017	1.000	no		Plant disease resistance response protein family protein.
*qPGWC8* (*qDEC8*)												
1	*Os08g0101500*	(−)2.64	−1.403	0.000	yes		(+)1.22	0.281	0.538	no	*OsSultr5;2*	Similar to sulfate transporter.
2	*Os08g0101800*	(−)2.80	−1.485	0.020	yes		(+)2.68	1.420	0.000	yes		Protein of unknown function DUF821, CAP10-like family protein.
3	*Os08g0104400*	9.27	−3.212	0.000	yes		(+)2.94	1.554	0.000	yes		Conserved hypothetical protein.
4	*Os08g0106100*	(−)5.57	−2.477	0.000	yes		(+)5793.33	12.500	0.000	yes		Conserved hypothetical protein.
5	*Os08g0111200*	(−)3.14	−1.652	0.007	yes	(−)2.87	(+)1.69	0.760	0.362	no		Beta-glucosidase, GBA2 type domain containing protein.
6	*Os08g0112300*	(−)3.54	−1.824	0.006	yes		(+)2.10	1.071	0.002	yes		Transferase domain containing protein.
7	*Os08g0113000*	(−)3.67	−1.877	0.006	yes		(+)2.43	1.279	0.000	yes	*prx117*	Similar to Peroxidase 47 precursor (EC 1.11.1.7) (Atperox P47) (ATP32).
8	*Os08g0114300*	(−)3.16	−1.660	0.000	yes		(+)1.62	0.699	0.008	no		D-arabinono-1,4-lactone oxidase domain containing protein.
9	*Os08g0114400*	(−)4.85	−2.277	0.000	yes		(+)1.88	0.912	0.003	no		Hypothetical protein.
10	*Os08g0116800*	(−)3.77	−1.913	0.016	yes		(+)4.95	2.309	0.155	no		Exoribonuclease domain containing protein.
11	*Os08g0120600*	(−)2.33	−1.219	0.005	yes	(−)5.67	(+)1.42	0.501	0.343	no		Similar to Fructose-bisphosphate aldolase, cytoplasmic isozyme (EC 4.1.2.13).
12	*Os08g0122800*	(−)6.45	−2.689	0.003	yes		(+)240.50	7.910	0.000	yes		Conserved hypothetical protein. (Os08t0122800-01); Kringle, conserved site domain containing protein.
13	*Os08g0124500*	(−)306.67	−8.261	0.018	yes		(+)96.67	6.595	0.114	no		Similar to Resistance protein candidate (Fragment); Similar to Resistance protein candidate (Fragment).

## Discussion

Cell division (cell number or cell size) is considered to contribute to the development and patterning of grain shape (Zhou et al. 2015b). Our finding in this study is that outer glume epidermal cell numbers and cell length were both significantly different between PYZX and P02428. In brief, the slender grains of PYZX were produced by longitudinally increasing cell length and cell number while transversely decreasing cell number (Fig. 2). The LWR of PYZX is more than twice the value of P02428, which to the best of our knowledge is the most extreme difference between parents of mapping populations used for QTL analysis of rice grain shape. Examination of the microstructures of rice endosperm of mature seeds demonstrated that the arrangement of the endosperm of PYZX was more compact than that of P02428, which exhibited starch granules with more spherical surfaces and uniform size. The differences in the starch granule shape and the arrangement of the granules resulted in the higher PGWC and DEC percentage in P02428, which was in agreement with previous research (Guo et al. 2011; Li et al. 2014b). The PGWC and DEC parameters were related to multiple investigated grain shape traits, and gave a maximum correlation coefficient with grain width (Table 1). These results were in accordance with Adu-Kwarteng et al. (2003); Zhou et al. (2015a), considering grain width had positive and high correlation with chalkiness. Starch granules in translucent areas of grains are bigger and more tightly packed than the small loosely packed granules in chalky areas of the grain (Lisle et al. 2000), and the hypothesis is that source-sink interactions involved in grain-filling are involved in the formation of chalk. Hence, the processes of starch synthesis were the focus of many studies about grain chalk (Fitzgerald et al. 2009). Previous studies demonstrate a complex mechanism for chalkiness formation in the rice endosperm. Although many starch-metabolic genes have been characterized in the rice mutants, few corresponding to the QTLs for grain chalkiness have been addressed (Sun et al. 2015). Our results provide valuable background information on the structural characteristics of hull and endosperm tissues, which facilitate the understanding of molecular mechanisms determining grain shape and chalkiness.

Since the advent of molecular markers, crop researchers and breeders have dedicated huge amounts of effort on QTL mapping in biparental populations and marker-assisted selection (MAS) (Chen et al. 2014a). High-throughput SNP genotyping and estimation of recombination points based on resequencing of recombinant inbred lines were recently utilized, even though the sequencing coverage was insufficient (Huang et al. 2009; Xie et al. 2010). In this study, a total of 85,743 high-quality population SNPs with even distribution throughout the entire genome were detected using GBS strategy. Recombination breakpoints were determined by checking the positions where genotypes change. By this way, raw SNPs were converted into effective recombination bin, and these small recombination bins can be regarded as an effective type of genetic marker (Wang et al. 2011). The number of bin makers (a total of 2711) of the linkage map were increased significantly, compared to the previous study (1495 in total) using the RICE6K SNP array, which mapped QTLs of grain shape using a similar population (197 RILs derived from the cross between indica variety ZS97 and japonica variety XZ2) (Hu et al. 2013). It is generally considered that the efficiency of QTL mapping largely depends on marker density and QTL mapping resolution can be improved with greater marker density to detect the locations of recombination events more precisely (Pan et al. 2012; Chen et al. 2014b). Yu et al. (2011) detected QTL using an ultra-high density SNP map based on population sequencing relative to traditional RFLP/SSR markers, and indicated that compared to RFLP/SSR and array-based SFP genotyping methods, the sequence-based method produces a map of the highest density, while the accuracy and the quality of the SNP markers was enhanced by using information of adjacent SNPs to form bins. In our study, take the example of QTLs of PGWC, the genetic intervals range from 0.26 to 5.66 cM, with an average of 2.14 cM, while the QTL clusters of PGWC were delimited into a physical region of few hundred kb, which had narrowed significantly compared to previous QTL mapping studies about grain chalkiness with SSR (Mei et al. 2013; Peng et al. 2014; Zhao et al. 2016b).

In general, it was expected that more QTLs for target traits with a mapping population derived from two contrasting parents would be detected (Bai et al. 2010). In our study, the mapping population derived from PYZX and P02428 showed an extremely wide diversity in rice grain shape and chalkiness traits, thus it was ideal for identification of main and minor effect QTL. Using single environment analysis, 109 and 27 QTLs associated with grain shape and chalkiness were detected respectively. Among these, 58 (53.2 %) of the QTLs for grain shape were detected in two or more environments whereas only 6 (22.2 %) of the chalkiness-related QTLs were observed in more than one of the environments (Additional file 1: Table S3). This confirmed that chalkiness was considerably affected by environment and exhibited a pattern of instability, whereas rice grain shape was fixed as long as the panicle was normally differentiated and mainly controlled by genotype and had higher heritability (Bai et al. 2010). *GS3*, which is reported as a major QTL for grain length and weight and a minor QTL for grain width and thickness in rice, encodes a putative transmembrane protein. It was located on the *qGS3.2* region in our QTL analysis, which was recurrent across four environments for GL, LWR, CS, PL and AS, showing the most stable expression and pleiotropic effects (Fan et al. 2006). The reported QTL of *GL7* (Wang et al. 2015b) regulates longitudinal cell elongation and results in an increase in grain length and improvement of grain appearance quality. In our study, *GL7* was detected in the QTL region of *qGS7.2* (associated with CS, GW, LWR, PL and GL) and also displayed consistent heredity and pleiotropism. Similarly, we detected a new QTL cluster of *qGS5.2* associated with GW, LWR, CS and AS with high PVE levels and stable performance across multiple environments, representing potential targets for gene cloning. In addition, there were some new-found important QTL clusters, such as *qGS3.1* associated with AS, PL, CS, GL and LWR, *qGS7.1* associated with CS, LWR, GL and PL, and *qPGWC8* (*qDEC8*) associated with PGWC and DEC, which offer an opportunity for improving grain appearance in rice for the future. As the main grain quality related traits, we intend to develop backcrossing introgression lines of the novel QTLs in the background of elite high-yielding varieties which should be useful to improve the grain quality. “Consumer-Targeted Rice Breeding” is emphasized especially in the rice quality area, and the grain shape QTLs should been utilized based on the specificity of consumer preference (Calingacion et al. 2014). The stable QTLs across multiple environments gained in this study enable incorporation of favorable alleles into agronomically superior germplasm by using technology of MAS combined with backcrossing method. Moreover, grain appearance quality had a close relation with milling quality (Lou et al. 2009). And milling yield of short and medium grain rice is typically higher than long grain rice (Kepiro et al. 2008). QTLs associated to more grain shape parameters (including GW, GL, LWR, CS, PL, and AS) had been analysis in this study, and different effects of the alterable combinations of target QTLs could probably strike a balance between grain shape and milling trait. In our practice, MAS have being performing by introgression of multiple QTL alleles of low chalk and grain length (preferred by local consumers of south China) into the elite restorer lines (R) and maintainer lines (B) in our lab, with the aim of improving the grain quality characters in hybrid rice.

Another advantageous feature of our study was the integration of the QTL mapping of chalkiness-related traits and the transcriptome profiling. Obtaining gene expression information using bulked RIL pools is simple, effective and aim-focused (Kloosterman et al. 2010), making it possible to assay a large number of our subjects. Hence it became possible to identify and narrow down our search for putative candidate genes affecting chalkiness-related traits. By this analysis, several differentially expressed genes in the three QTL intervals on chromosomes 5, 7 and 8 appear to have high probability of the target genes, considering their protein function prediction information as well. For instance, bidirectional sugar transporter *SWEET3a*, which was identified to be a DEG located on the region of *qPGWC5* (*qDEC5*), is involved in carbohydrate transmembrane transport process (http://www.ebi.ac.uk/QuickGO/) and predicted to mediate both low-affinity uptake and efflux of sugar across the plasma membrane (http://string-db.org/newstring_cgi/). The major up-regulated expression observed in P02428 is highly likely to influence carbohydrate transport and result in high levels of chalkiness. In another example, *Os07g0604800* (*OsUAM3*) which co-located with the *qPGWC7* (*qDEC7*) QTL in our study and the previously reported *qPGWC7* QTL (Zhou et al. 2009) was detected to be sharply down-regulated in the L-Pool and PYZX compared to the H-Pool and P02428. *OsUAM3* gene has an annotated as having UDP-arabinopyranose mutase activity (http://www.shigen.nig.ac.jp/rice/oryzabase/) and was predicted to be analpha-1,4-glucan-protein synthase and UDP-glucose/protein transglucosylase 1 (http://rapdb.dna.affrc.go.jp/). Given that carbohydrate metabolism is considered to play an important role in endosperm chalkiness (Wang et al. 2008), these DEGs represent strong candidates for genes underlying these chalkiness-associated QTLs. For instance, the rice *GIF1* (GRAIN INCOMPLETE FILLING 1) gene is required for carbon partitioning during early grain-filling and the *gif1* mutant grains showed more chalkiness, which accumulated lower levels of glucose and fructose, as well as sucrose (Wang et al. 2008). The *UGPase1* gene played a key role in seed carbohydrate metabolism and inactivation of the *UGPase1* gene caused endosperm chalkiness in rice (Woo et al. 2008). The several DEGs we identified here were involved in carbohydrate synthesis or transportation, indicating their potential role in the formation of endosperm chalkiness. Our identification of 668 functionally annotated DEGs between bulked RIL pools (Additional file 3: Table S5) provides a basis for dissecting the regulatory network governing the chalkiness trait. While detailed insight into these genes will be of great importance in unraveling the complex nature of rice chalkiness and also in elucidating the role of the diverse QTLs involved. The integrated approach utilizing the identified stable QTLs and transcriptome profiling could serve as a platform for candidate gene identification for genetic dissection and provide basal tools for molecular breeding in rice.

## Conclusion

Using the Genotyping-By-Sequencing approach, a genetic linkage map with an average distance of 0.865 cM between adjacent markers was constructed based on a RIL population in rice. A global mapping of quantitative trait loci affecting the grain shape and chalkiness traits were detected on four environments and the stable QTL clusters were highlighted and analyzed. The results of the transcriptome analysis demonstrated an available gene expression profile responsible for the development of chalkiness, and several important differentially expressed genes were co-located on the chalkiness-related QTL regions on chromosomes 5, 7, and 8. Critical loci were investigated and identified as candidate genes, which were suitable for functional validation and breeding utilization.

## Methods

### Plant material

In this study, the mapping population consisting of 192 recombinant inbred lines (RILs) derived by single-seed descent from an inter-subspecific cross of the indica PYZX and the japonica P02428, was used to perform QTL analyses for grain shape and chalkiness traits. RILs and the parental lines were field planted in Guangzhou (traditional flatland field) and Zengcheng (low hill district) in Guangdong province of China at the dry season (DS) in 2014 and the wet season (WS) in 2015, which have been named G-DS, Z-DS, G-WS, and Z-WS respectively in this study.

### Morphological and cellular analyses

Paraffin-embedded sections of spikelet samples were prepared according to Kim et al. (2014), with minor modifications. The materials were fixed in FAA and stored at 4 °C for 24 h. The fixed spikelets were dehydrated in a gradient ethanol series, and were then incubated in 100 % ethanol overnight. Dehydrated spikelets were embedded in Paraplast Plus (Sigma). The transverse sections of each spikelet were stained with 0.5 % toluidine blue, and viewed using an SZX10 stereomicroscope (Olympus, Tokyo, Japan). For scanning electron microscopy analysis, spikelet hull and endosperm samples were processed according to Wang et al. (2015b) and Li et al. (2014b). Young spikelets were fixed in 4 % (w/v) paraformaldehyde and 0.25 % glutaraldehyde in 0.1 M sodium phosphate buffer, pH 7.2, at 4 °C overnight. Fixed spikelets were dehydrated in a graded ethanol series, and 100 % ethanol was replaced with 3-methylbutyl acetate (Toriba et al. 2010). Milled rice grains were transversely cut in the middle with a knife and were coated with gold under vacuum conditions. Samples were dried at their critical point, sputter coated with platinum, and observed with the XL-30-ESEM instrument at an accelerating voltage of 5 kV.

### Evaluation of grain shape and chalkiness traits

Images of the mature grain were captured on a CanoScan 5600 F (Canon, Japan) scanner with the supplied software without image enhancement, and the grain shape parameters of GW, GL, LWR, CS, PL, and AS were measured precisely using SmartGrain Software (Tanabata et al. 2012). The chalkiness parameters were measured with an automatic machine JMWT 12 according to Xu et al. (2012). Two metrics were used to describe grain chalkiness as previously described (Zhao et al. 2016a): percentage of grains with chalk (PGWC) and degree of endosperm chalkiness (DEC) which is the ratio of total chalky area to the total kernel area of all sampled grains. The statistical analysis was performed with SPSS statistics 18.0 and Microsoft Excel.

### DNA extraction, genotyping by sequencing, and SNP identification

Leaf samples were collected from two parental lines and 192 RILs at F_7_ generation. DNAs were extracted using the CTAB method and quantified using both a NanoDrop ND-1000 Spectrophotometer and agarose gel electrophoresis. In our study, the genome of parental lines, PYZX and P02428, were directly sequenced to ~25× coverage, while the RILs were subjected to Genotyping By Sequencing (GBS) as described by Elshire et al. (2011). The DNA samples of the RIL population digested using *Mse*I and *Hae*III. The other basic schematic of the protocol used for performing GBS was according to Duan et al. (2013).

Sequencing was performed on the Illumina HiSeq 2500 platform to generate 150 bp paired-end reads (Novogene Bioinformatics Technology Co., Ltd, China). The original image data generated were converted into sequence data via base calling (Illumina pipeline CASAVA v1.8.2) and then subjected to the quality control (QC) procedure to remove unusable reads: 1) reads contain the Illumina library construction adapters; 2) reads contain more than 10 % unknown bases (N bases); 3) one end of a read contains more than 50 % low quality bases 4) Sequencing reads were aligned to the reference genome (http://plants.ensembl.org/Oryza_sativa/) using BWA with default parameters. Subsequent processing, including duplicate removal was performed using SAMtools and PICARD (http://picard.sourceforge.net). The raw SNP/InDel sets are called by SAMtools with the parameters as ‘-q 1 -C 50 –m 2 -F 0.002 -d 1000’. Then we filtered these sets using the following criteria: (1) mapping quality >20; (2) depth of the variant position >4.

### Bin marker production and QTL analysis

To overcome the false positive of SNPs genotype of the population, the sliding window approach adopted by Huang et al. (2009) with some modification was used to evaluate a group of consecutive SNPs for genotyping. The genotypic maps of the RILs were aligned and split into recombination bins according to the recombination breakpoints, with the parameter of window size of 15 SNPs. Bins less than 300-kb were merged with the next bin. Genotypes of bins for regions at the transitions between two different genotype blocks were set to missing data. Segregation distortion markers showing distorted segregation (*P* < 0.01) were discarded. For this step, a total of 85,743 high-quality SNPs were used for bin map construction, containing 2711 bin markers.

SNP bin markers were used to construct the genetic linkage map using the est.map function of the R/qtl package (Broman et al. 2003) with the Kosambi map method, and the marker genetic distances were estimated. The QTLs were mapped with the inclusive composite interval mapping method using the QTL IciMapping software version 4 (Li et al. 2007; Meng et al. 2015) with single-environment phenotypic values. QTLs were calculated using the ICIM-ADD mapping method, with mapping parameters of 1 cM step and 0.001 probabilities in a stepwise regression. The threshold for logarithm of odds (LOD) scores was set as 2.5, and the QTLs in a particular genomic region with the LOD values larger than this threshold were called (Li et al. 2014a). The regional genes were annotated and analyzed via the database of RAP (http://rapdb.dna.affrc.go.jp) and Ensembl (http://plants.ensembl.org/Oryza_sativa).

### Experimental design for transcriptome profiling study

To obtain an overview of the transcriptome profiling and differential gene expression pattern relating to the chalky trait, two bulked RIL pools with extreme tails of the chalky trait were developed, consisting of 13 bulks RIL individuals respectively. The L-Pool was constructed from individuals with extremely low levels at PGWC and DEC, conversely the H-Pool bears high levels of PGWC and DEC parameters (the details are available in Additional file 1: Table S6). The pools were used for RNA-Seq analysis along with the parental lines. After approximately 20 days after fertilization, grain samples of each group were collected and stored at −80 °C in preparation for RNA-Seq.

### RNA isolation, sequencing and statistical analysis of gene expression profile

The total RNA of each of the above listed samples was homogenized using mortar and pestle with liquid nitrogen and purified using the Plant Total RNA Purification Kit (Dakewe Biotech Company) following the manufacturer’s instructions. RNA quality was verified using Agilent 2100 Bio-analyzer (Agilent Technologies, Santa Clara, CA) and was also checked by RNase free agarose gel electrophoresis. Next, Poly (A) mRNA was isolated using oligo-dT beads (Qiagen). All mRNA was broken into short fragments by adding fragmentation buffer. First-strand cDNA was generated using random hexamer-primed reverse transcription, followed by the synthesis of the second-strand cDNA using RNase H and DNA polymerase I. The cDNA fragments were purified using a QIAquick PCR extraction kit. The cDNA library was sequenced on the Illumina sequencing platform (IlluminaHiSeq™ 2500) using the paired-end technology by Gene Denovo Co. (Guangzhou, China). A Perl program was written to select clean reads by removing low quality sequences (there were more than 50 % bases with quality lower than 20 in one sequence), reads with more than 5 % N bases (bases unknown) and reads containing adaptor sequences. Sequencing reads in FASTQ format were mapped to the reference genome (http://plants.ensembl.org/Oryza_sativa/) and splice junctions were identified using TopHat (Kim et al. 2013). The Cufflinks package (Trapnell et al. 2012) was used for genome guided transcript assembly and the expression abundance was estimated.

After the expression level of each transcript and gene was calculated, differential expression analysis was conducted using edgeR (Robinson et al. 2010). The false discovery rate (FDR) was used to determine the threshold of the *p* value in multiple tests, and for the analysis, a threshold of the FDR ≤ 0.05 and an absolute value of log_2_Ratio ≥ 1 were used to judge the significance of the gene expression differences. The differentially expressed genes were used for GO (Gene Ontology) and KEGG (Kyoto Encyclopedia of Genes and Genomes) enrichment analyses according to a method similar to that described by Zhang et al. (2013). Both GO terms and KEGG pathways with a Q-value ≤0.05 are significantly enriched in DEGs. To compare the differential gene expression between PYZX versus P02428, and between bulked RIL pools, we took PYZX and the H-pool as baseline controls, respectively.

### Validation of gene expression by qRT–PCR

Expression levels of five genes (*Os05t0214300-00*, *Os05t0215300-01*, *Os07t0604800-01*, *Os08t0101500-01*, and *Os08t0120600-01*) were selected for the validation of RNA-seq results using quantitative real-time PCR (qRT-PCR). The mRNA sequences of the five genes were downloaded from EnsemblPlants database (http://plants.ensembl.org/Oryza_sativa/), and were used for primers design using Primer3 software. The primer sequences are listed in Additional file 2: Table S4. First strand cDNA was prepared from 2 μg of total RNA in 50 μl of reaction volume using the high-capacity cDNA Archive kit (Applied Biosystems, USA). Two μl of the first strand cDNA reaction was used for quantitative real time PCR. qRT-PCR was conducted using the AceQ qPCR SYBR Green Master Mix Kit (Vazyme Biotech) according to standard protocol, and the expression levels of the genes were determined on the StepOnePlus System (Applied Biosystems, USA). Three biological and three technical replicates were taken for each treatment. As an endogenous control, *Actin* was used for the normalization of Ct value obtained and the relative expression values were calculated by ΔΔCt method.

## Additional files

Additional file 1:
**Table S1.** Comparison of eight traits between PYZX, P02428 and RILs in four environments in this study. **Table S2.** Annotation statistics of the SNPs between PYZX and P02428. **Table S3.**QTLs detected in the 192 RIL lines derived from the cross between PYZX and P02428 in four environments. **Table S6.**Details of PGWC and DEC parameters of the L-Pool and H-Pool. **Table S7.**Primers used for qRT-PCR. (PDF 86 kb) Additional file 2:
**Table S4.** Annotated function of differentially expressed genes identified between parents. (XLSX 1232 kb) Additional file 3:
**Table S5.** Annotated function of differentially expressed genes identified between RIL segregant pools. (XLSX 170 kb) Additional file 4:
**Figure S1.** GO (Gene Ontology) Enrichment of differentially expressed genes. (PPTX 188 kb)

## Abbreviations

AS: Area size of grain
CS: Circularity
DEC: Degree of endosperm chalkiness
DEG: Differentially expressed gene
DS: Dry season
GBS: Genotyping-by-sequencing
GL: Grain length
GW: Grain width
LWR: The ratio of grain length and width
PGWC: Percentage of grain with chalkiness
PL: Perimeter length of grain
WS: Wet season
