# Supplementary material for: High-resolution QTL mapping for grain appearance traits and co-localization of chalkiness-associated differentially expressed candidate genes in rice
Source: Rice (N Y). 2016 Sep 22;9:48. doi: 10.1186/s12284-016-0121-6 (PMC5033801; doi:10.1186/s12284-016-0121-6)
Supplement: Additional file 1: — Table S1. Comparison of eight traits between PYZX, P02428 and RILs in four environments in this study. Table S2. Annotation statistics of the SNPs between PYZX and P02428. Table S3.QTLs detected in the 192 RIL lines derived from the cross between PYZX and P02428 in four environments. Table S6.Details of PGWC and DEC parameters of the L-Pool and H-Pool. Table S7.Primers used for qRT-PCR. (PDF 86 kb) [file 12284_2016_121_MOESM1_ESM.pdf]

Table S1. Comparison of eight traits between PYZX, P02428 and RILs under four environments in this study.

Traits	PYZX								P02428								RILs							
	G-DS		Z-DS		G-WS		Z-WS		G-DS		Z-DS		G-WS		Z-WS		G-DS		Z-DS		G-WS		Z-WS	
	Mean	S.D.	Mean	S.D.	Mean	S.D.	Mean	S.D.	Mean	S.D.	Mean	S.D.	Mean	S.D.	Mean	S.D.	Mean	S.D.	Mean	S.D.	Mean	S.D.	Mean	S.D.
GW (mm)	2.233	0.015	2.263	0.015	2.227	0.025	2.320	0.010	3.790	0.056	3.720	0.079	3.653	0.042	3.853	0.076	2.765	0.019	2.752	0.020	2.638	0.020	2.701	0.020
GL (mm)	12.293	0.076	12.303	0.076	11.937	0.180	12.143	0.071	7.300	0.303	7.407	0.117	6.977	0.129	6.967	0.250	9.543	0.059	9.451	0.059	9.215	0.061	9.172	0.059
LWR	5.503	0.015	5.437	0.015	5.360	0.062	5.233	0.047	1.927	0.086	1.990	0.056	1.623	0.488	1.807	0.100	3.491	0.038	3.439	0.039	3.541	0.041	3.473	0.037
CS	0.363	0.015	0.370	0.015	0.363	0.006	0.363	0.021	0.730	0.010	0.727	0.006	0.720	0.010	0.740	0.010	0.513	0.004	0.516	0.004	0.506	0.004	0.518	0.004
AS (mm2)	21.407	0.187	22.140	0.187	20.107	0.127	21.300	0.935	19.847	0.220	19.997	0.614	18.583	1.535	19.273	0.420	20.120	20.120	19.846	0.154	18.436	0.149	18.791	0.155
PL (mm)	27.150	0.217	27.493	0.217	26.813	0.358	27.113	0.201	19.050	0.511	19.487	0.473	18.120	0.878	17.920	0.187	22.272	0.120	22.053	0.121	21.479	0.122	21.438	0.121
PGWC (%)	1.450	0.391	1.000	0.391	0.333	0.577	1.000	1.000	87.000	2.000	88.333	4.041	95.333	3.786	96.000	2.646	26.706	2.231	24.639	2.205	41.442	2.690	37.039	2.511
DEC	0.14	0.08	0.09	0.08	0.04	0.07	0.08	0.08	50.60	1.13	50.50	1.85	57.20	1.25	58.33	1.05	10.16	0.89	26.10	1.04	14.64	1.19	15.36	1.27

Table S2. Annotation statistics of the SNP between PYZX and P02428.

Category		Number of SNPs
Upstream		107753
Exonic	Stop gain	725
	Stop loss	272
	Synonymous	36076
	Non-synonymous	43509
Intronic		123531
Splicing		171
Downstream		92323
upstream/downstream		17332
Intergenic		1112344
Total		1534036

Table S3. QTLs detected in the 192 RIL lines derived from the cross between PYZX and P02428 on four environments.

ID	Trait	Environment	Chromosome	Position (cM)	Nearest Left Marker	Nearest Right Marker	LOD	PVE (%)	Add
1	Grain width	G-DS	1	140	mk157	mk158	4.230	9.753	0.087
2	Grain width	G-WS	1	140	mk157	mk158	3.889	6.346	0.069
3	Grain width	G-DS	1	209	mk275	mk276	7.410	9.636	-0.081
4	Grain width	G-WS	1	229	mk289	mk290	4.948	7.943	-0.077
5	Grain width	Z-WS	1	232	mk294	mk295	3.761	7.422	-0.076
6	Grain width	G-WS	3	20	mk575	mk576	3.565	5.492	-0.066
7	Grain width	Z-DS	4	111	mk994	mk995	4.417	9.119	-0.082
8	Grain width	G-DS	5	35	mk1174	mk1175	7.396	10.023	-0.082
9	Grain width	G-WS	5	35	mk1174	mk1175	3.252	5.000	-0.061
10	Grain width	Z-DS	5	61	mk1190	mk1191	5.783	12.377	-0.095
11	Grain width	G-WS	5	62	mk1191	mk1192	5.298	10.936	-0.092
12	Grain width	G-WS	5	80	mk1225	mk1226	2.895	4.691	-0.060
13	Grain width	G-DS	5	98	mk1240	mk1241	2.711	3.354	-0.048
14	Grain width	G-DS	7	131	mk1743	mk1744	9.209	12.326	-0.091
15	Grain width	G-WS	7	131	mk1743	mk1744	6.511	10.313	-0.087
16	Grain width	G-DS	8	99	mk1895	mk1896	4.678	6.002	0.064
17	Grain width	Z-WS	9	100	mk2073	mk2074	4.459	8.899	0.082
18	Grain length	Z-WS	2	81	mk380	mk381	3.033	5.936	0.193
19	Grain length	Z-DS	2	90	mk401	mk402	4.781	8.689	0.235
20	Grain length	G-DS	2	91	mk404	mk405	4.654	6.103	0.199
21	Grain length	Z-WS	3	152	mk690	mk691	6.200	12.401	0.290
22	Grain length	Z-DS	3	152	mk690	mk691	5.856	10.967	0.276
23	Grain length	G-DS	3	153	mk691	mk692	12.252	18.042	0.357
24	Grain length	G-WS	3	155	mk693	mk694	12.762	20.423	0.396
25	Grain length	Z-DS	3	244	mk794	mk795	2.648	4.662	0.175
26	Grain length	G-DS	3	275	mk819	mk820	3.891	5.751	0.193
27	Grain length	G-WS	3	275	mk819	mk820	5.690	8.828	0.244
28	Grain length	Z-WS	3	277	mk821	mk822	2.921	5.600	0.190
29	Grain length	G-WS	4	260	mk1107	mk1108	4.526	6.409	0.206
30	Grain length	G-DS	6	181	mk1534	mk1535	2.700	3.904	0.158
31	Grain length	G-WS	7	128	mk1738	mk1739	11.568	17.573	0.344
32	Grain length	G-DS	7	132	mk1744	mk1745	13.890	20.799	0.366
33	Grain length	G-DS	12	72	mk2581	mk2582	2.985	4.030	0.160
34	The ratio of grain length and width	Z-WS	1	229	mk289	mk290	3.436	6.611	0.135
35	The ratio of grain length and width	G-DS	1	230	mk291	mk292	3.710	4.796	0.113
36	The ratio of grain length and width	Z-DS	2	90	mk401	mk402	3.138	5.755	0.118
37	The ratio of grain length and width	G-DS	2	91	mk404	mk405	2.765	3.540	0.096
38	The ratio of grain length and width	Z-WS	3	150	mk690	mk691	3.598	7.283	0.147

39	The ratio of grain length and width	Z-DS	3	152	mk690	mk691	3.789	7.239	0.139
40	The ratio of grain length and width	G-DS	3	155	mk693	mk694	6.391	8.714	0.162
41	The ratio of grain length and width	G-WS	3	155	mk693	mk694	7.940	12.643	0.211
42	The ratio of grain length and width	Z-DS	3	219	mk782	mk783	2.933	5.286	0.119
43	The ratio of grain length and width	G-DS	3	244	mk794	mk795	3.674	4.876	0.115
44	The ratio of grain length and width	G-DS	5	35	mk1174	mk1175	6.801	9.463	0.157
45	The ratio of grain length and width	Z-DS	5	64	mk1196	mk1197	3.379	6.191	0.123
46	The ratio of grain length and width	Z-WS	5	64	mk1196	mk1197	4.469	8.613	0.153
47	The ratio of grain length and width	Z-DS	7	123	mk1734	mk1735	4.297	8.141	0.140
48	The ratio of grain length and width	G-DS	7	131	mk1743	mk1744	13.879	20.482	0.231
49	The ratio of grain length and width	G-WS	7	132	mk1744	mk1745	14.540	24.291	0.274
50	The ratio of grain length and width	G-WS	8	17	mk1803	mk1804	4.584	6.819	0.146
51	The ratio of grain length and width	Z-WS	9	100	mk2073	mk2074	3.427	6.598	-0.133
52	The ratio of grain length and width	G-DS	12	39	mk2541	mk2542	3.437	4.403	0.111
53	Circularity	G-WS	1	128	mk142	mk143	3.705	4.346	0.013
54	Circularity	Z-WS	1	231	mk292	mk293	4.201	7.673	-0.016
55	Circularity	G-DS	1	232	mk294	mk295	3.122	3.781	-0.010
56	Circularity	Z-DS	2	94	mk414	mk415	3.443	5.503	-0.013
57	Circularity	Z-WS	3	150	mk690	mk691	3.518	6.736	-0.016
58	Circularity	G-DS	3	155	mk693	mk694	7.256	9.370	-0.018
59	Circularity	G-WS	3	155	mk693	mk694	10.251	13.834	-0.024
60	Circularity	Z-DS	3	158	mk697	mk698	3.151	5.068	-0.013
61	Circularity	G-DS	3	244	mk794	mk795	4.559	5.740	-0.013
62	Circularity	G-WS	3	244	mk794	mk795	3.473	4.176	-0.012
63	Circularity	Z-DS	4	113	mk999	mk1000	6.313	10.189	-0.018
64	Circularity	Z-DS	4	205	mk1039	mk1040	3.382	5.308	0.013
65	Circularity	G-DS	5	35	mk1174	mk1175	8.912	11.617	-0.018
66	Circularity	G-WS	5	35	mk1174	mk1175	3.047	3.729	-0.011
67	Circularity	Z-WS	5	61	mk1190	mk1191	4.924	8.755	-0.017
68	Circularity	Z-DS	5	79	mk1223	mk1224	2.521	3.881	-0.011
69	Circularity	Z-DS	6	60	mk1380	mk1381	2.892	4.727	-0.012
70	Circularity	Z-DS	7	123	mk1734	mk1735	6.387	10.749	-0.018
71	Circularity	G-DS	7	131	mk1743	mk1744	16.025	22.466	-0.025
72	Circularity	G-WS	7	131	mk1743	mk1744	18.346	26.139	-0.030
73	Circularity	Z-WS	7	134	mk1746	mk1747	3.610	6.532	-0.015
74	Circularity	G-WS	8	17	mk1803	mk1804	2.875	3.403	-0.011
75	Circularity	Z-DS	9	98	mk2072	mk2073	2.524	3.592	0.010
76	Circularity	G-DS	12	39	mk2541	mk2542	4.395	5.274	-0.013
77	Area size of grain	G-DS	3	12	mk565	mk566	2.524	3.302	0.364
78	Area size of grain	G-WS	3	62	mk608	mk609	4.049	7.492	-0.551
79	Area size of grain	Z-DS	3	137	mk686	mk687	3.161	7.228	0.556
80	Area size of grain	Z-WS	3	138	mk686	mk687	3.883	7.981	0.586

81	Area size of grain	G-WS	3	139	mk688	mk689	8.125	16.135	0.806
82	Area size of grain	G-DS	3	140	mk689	mk690	10.032	14.595	0.757
83	Area size of grain	G-WS	4	2	mk868	mk869	2.616	5.040	-0.472
84	Area size of grain	G-DS	4	134	mk1003	mk1004	6.332	8.670	-0.598
85	Area size of grain	Z-DS	5	62	mk1191	mk1192	2.686	6.388	-0.530
86	Area size of grain	G-DS	5	70	mk1207	mk1208	4.387	5.971	-0.498
87	Area size of grain	Z-WS	5	101	mk1241	mk1242	4.619	10.546	-0.675
88	Area size of grain	Z-WS	5	135	mk1262	mk1263	4.081	8.842	0.620
89	Area size of grain	G-DS	6	178	mk1532	mk1533	3.534	4.876	0.439
90	Area size of grain	G-DS	7	21	mk1578	mk1579	6.167	8.879	0.639
91	Area size of grain	G-DS	10	75	mk2261	mk2262	7.044	10.657	0.651
92	Area size of grain	G-DS	11	213	mk2510	mk2511	4.149	5.861	0.481
93	Perimeter length of grain	G-WS	2	53	mk349	mk350	2.606	3.289	0.301
94	Perimeter length of grain	Z-WS	2	81	mk380	mk381	2.922	5.700	0.388
95	Perimeter length of grain	G-DS	2	90	mk401	mk402	4.330	6.003	0.399
96	Perimeter length of grain	Z-DS	2	90	mk401	mk402	4.789	8.674	0.480
97	Perimeter length of grain	Z-DS	3	149	mk689	mk690	6.179	11.464	0.563
98	Perimeter length of grain	Z-WS	3	152	mk690	mk691	6.379	12.763	0.604
99	Perimeter length of grain	G-DS	3	153	mk691	mk692	12.449	18.648	0.737
100	Perimeter length of grain	G-WS	3	155	mk693	mk694	14.296	21.024	0.813
101	Perimeter length of grain	G-WS	3	244	mk794	mk795	5.079	6.469	0.430
102	Perimeter length of grain	G-DS	3	275	mk819	mk820	3.743	5.557	0.385
103	Perimeter length of grain	Z-WS	3	277	mk821	mk822	2.956	5.665	0.392
104	Perimeter length of grain	G-DS	4	215	mk1044	mk1045	2.515	3.484	-0.304
105	Perimeter length of grain	G-WS	4	263	mk1110	mk1111	4.082	5.110	0.373
106	Perimeter length of grain	G-DS	6	181	mk1534	mk1535	3.140	4.608	0.348
107	Perimeter length of grain	G-WS	7	128	mk1738	mk1739	9.319	12.409	0.584
108	Perimeter length of grain	G-DS	7	133	mk1745	mk1746	12.403	18.442	0.701
109	Perimeter length of grain	G-WS	10	74	mk2261	mk2262	3.290	4.021	0.333
110	Perimeter length of grain	G-DS	12	72	mk2581	mk2582	2.726	3.710	0.312
111	Percentage of grain with chalkiness	Z-DS	2	168	mk473	mk474	3.165	5.594	-7.291
112	Percentage of grain with chalkiness	Z-DS	4	113	mk999	mk1000	7.035	12.975	-10.997
113	Percentage of grain with chalkiness	G-DS	4	135	mk1005	mk1006	5.635	9.738	-9.579
114	Percentage of grain with chalkiness	G-WS	5	13	mk1154	mk1155	2.638	4.971	-8.125
115	Percentage of grain with chalkiness	G-DS	5	35	mk1174	mk1175	5.250	8.985	-8.999
116	Percentage of grain with chalkiness	Z-DS	5	60	mk1189	mk1190	6.347	11.890	-10.337
117	Percentage of grain with chalkiness	G-WS	5	60	mk1189	mk1190	4.007	8.143	-9.706
118	Percentage of grain with chalkiness	G-WS	5	84	mk1228	mk1229	2.773	5.182	-8.332
119	Percentage of grain with chalkiness	G-WS	6	55	mk1378	mk1379	3.002	5.819	-8.807
120	Percentage of grain with chalkiness	Z-DS	6	78	mk1408	mk1409	2.568	4.457	-6.255
121	Percentage of grain with chalkiness	Z-DS	7	132	mk1744	mk1745	3.662	7.123	-7.934
122	Percentage of grain with chalkiness	G-DS	7	134	mk1746	mk1747	2.946	4.763	-6.593

123	Percentage of grain with chalkiness	Z-WS	8	1	mk1787	mk1788	4.804	9.866	-10.693
124	Percentage of grain with chalkiness	G-DS	8	4	mk1790	mk1791	4.076	6.701	-7.893
125	Percentage of grain with chalkiness	G-WS	8	17	mk1803	mk1804	4.580	8.645	-10.740
126	Percentage of grain with chalkiness	Z-WS	9	108	mk2078	mk2079	2.642	5.430	-7.888
127	Degree of endosperm chalkiness	Z-WS	4	110	mk991	mk992	3.016	6.342	-3.745
128	Degree of endosperm chalkiness	G-DS	4	136	mk1006	mk1007	3.779	7.760	-2.188
129	Degree of endosperm chalkiness	G-WS	5	18	mk1160	mk1161	3.589	5.831	-4.473
130	Degree of endosperm chalkiness	G-DS	5	35	mk1174	mk1175	3.354	6.847	-2.014
131	Degree of endosperm chalkiness	Z-DS	5	60	mk1189	mk1190	3.660	8.462	-1.813
132	Degree of endosperm chalkiness	G-WS	5	61	mk1190	mk1191	3.516	7.464	-4.003
133	Degree of endosperm chalkiness	Z-WS	6	23	mk1339	mk1340	2.516	5.391	-3.390
134	Degree of endosperm chalkiness	G-WS	6	64	mk1387	mk1388	7.378	12.779	-6.611
135	Degree of endosperm chalkiness	G-WS	8	6	mk1792	mk1793	6.002	10.074	-5.931
136	Degree of endosperm chalkiness	G-DS	8	130	mk1924	mk1925	3.411	7.027	-2.046

Table S6. Details of PGWC and DEC parameters of the L-Pool and H-Pool.

	lines	PGWC (%)		DEC
L-Pool	GL25	4.05		1.30
	GL40	4.07		0.60
	GL48	2.13		1.60
	GL57	2.19		0.20
	GL73	4.06		2.90
	GL97	1.20		1.90
	GL104	8.21		1.51
	GL108	3.07		1.20
	GL109	0.00		0.00
	GL117	2.01		0.00
	GL167	5.19		1.50
	GL168	4.31		0.00
	GL169	7.19		3.10
H-Pool	GL19	100.00		97.30
	GL32	100.00		99.20
	GL47	100.00		96.40
	GL81	98.02		95.80
	GL82	98.12		97.70
	GL110	100.00		95.30
	GL134	100.00		98.60
	GL139	99.21		97.00
	GL141	99.02		97.90
	GL144	99.03		89.60
	GL150	100.00		99.30
	GL156	100.00		98.50
	GL162	98.17		98.00

Table S7. Primers used for qRT-PCR.

Gene ID	Forward Sequence	Reverse Sequence	Product Size (bp)
Os05t0214300-00	ATGGTTTTCCTGTGGTGAGC	GATGCCATCAGCATGACTTG	148
Os05t0215300-01	GATGAATGCAGCAATGATGG	GAGTTCACCAATCCGCATCT	147
Os07t0604800-01	TACATCTGGCACAGCAAAGC	CTTGGCCAGCTCAAGGTAAC	156
Os08t0111200-01	TCACCGTTCATTCCTCACAA	TCCCGGTCAGTTCAGATTTC	151
Os08t0120600-01	GCCTACTGCGGCAAGTACA	CGGTTCTCCTCGACATTCTC	149
Acting	CCCTCCTGAAAGGAAGTACAGTGT	GTCCGAAGAATTAGAAGCATTTCC	146
