# Supplementary material for: High-resolution QTL mapping for grain appearance traits and co-localization of chalkiness-associated differentially expressed candidate genes in rice
Source: Rice (N Y). 2016 Sep 22;9:48. doi: 10.1186/s12284-016-0121-6 (PMC5033801; doi:10.1186/s12284-016-0121-6)
Supplement: Additional file 4: — Figure S1. GO (Gene Ontology) Enrichment of differentially expressed genes. (PPTX 188 kb) [file 12284_2016_121_MOESM4_ESM.pptx]

## Slide 1
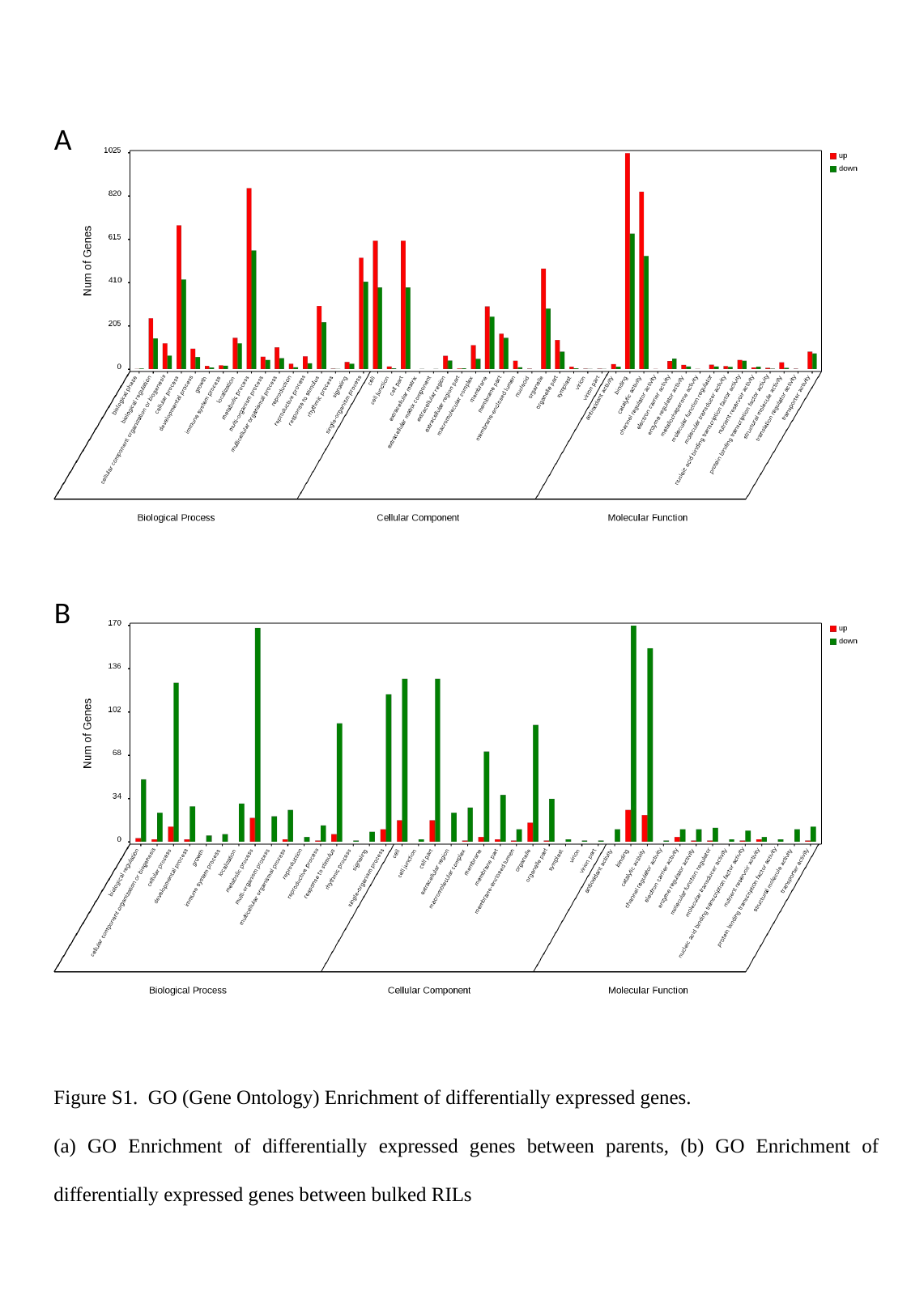

A
B
Figure S1.  GO (Gene Ontology) Enrichment of differentially expressed genes.
(a) GO Enrichment of differentially expressed genes between parents, (b) GO Enrichment of differentially expressed genes between bulked RILs
